# Synthesis, Characterization, and X-ray Crystallography, of the First Cyclohexadienyl Trifluoromethyl Metal Complex (η^5^-C_6_H_7_)Fe(CO)_2_CF_3_

**DOI:** 10.3390/molecules27217595

**Published:** 2022-11-05

**Authors:** Chris Douvris, David Matatov, Derek Bussan, Christos Lampropoulos, Donald J. Wink

**Affiliations:** 1 Department of Biological and Chemical Sciences, New York Institute of Technology, Old Westbury, NY 11564, USA; 2 Department of Chemistry, Eastern Kentucky University, 521 Lancaster Ave., Richmond, KY 40475, USA; 3 Florida State College at Jacksonville, Jacksonville, FL 32202, USA; 4 James Weldon Johnson MS, Duval County Public Schools, Jacksonville, FL 32207, USA; 5 Department of Chemistry, University of Illinois Chicago, 845 W. Taylor St., Chicago, IL 60607, USA

**Keywords:** Fe complexes, trifluormethyl, cyclohexadienyl, nucleophilic trifluoromethylation

## Abstract

Fluorochemistry is a field of tremendous developments and advances in several areas of science including materials, pharmaceuticals and agriculture. This makes the design and synthesis of fluorine-containing substances highly desirable research targets. The sub-area of synthetic perfluorinated chemistry proportionately attracts widespread interest by applying to all areas of chemistry including organic and inorganic. Particularly, the latter is much underdeveloped as metal complexes with perfluoroalkyl moieties are scarce, with the vast majority of perfluorinated analogs, of long known, halo and alkylated derivatives never having been synthesized. Focusing on the chemistry of trifluoromethyl group, which is the most important in the class of perfluoroalkyls, we set out to explore the possibility of synthesizing and completely characterizing a cyclohexadienyl metal complex. Upon utilizing a number of trifluorometylating reagents, we only arrived at an efficient preparation by the use of Morrison’s trifluormethylating reagent. As a result, the new, air- and moisture-sensitive complex (η^5^-C_6_H_7_)Fe(CO)_2_CF_3_, was prepared in 71% yield, using a nucleophilic iodo-for-trifluoromethyl substitution, and was completely characterized including by X-ray crystallography.

## 1. Introduction

The chemistry and applications of the trifluoromethyl group have a special place in fluorine chemistry as they continue to intrigue and influence both academic research and industrial laboratories [1,2]. On one hand, academic researchers are attracted by the variety of interesting properties of the CF_3_ moiety, including its high electronegativity, CF_3_-induced solubility changes, C-F activation, including C-F oxidative addition and reductive elimination, as well. ^19^F NMR spectroscopy monitoring of molecules and intermediates [3,4]. On the other hand, the industry is heavily utilizing the trifluoromethyl moiety for the synthesis of a variety of pharmaceuticals and drugs, often replacing a chloride or a methyl group [5,6]. An illustration of this point is the fact that several notable drugs contain trifluoromethyl groups including the antidepressant Prozac, the HIV reverse transcriptase inhibitor Sustiva, and the nonsteroidal anti-inflammatory drug, Celebrex [7].

In organometallic chemistry, despite the high interest on the CF_3_ moiety, the synthesized trifluoromethylated metal complexes are considerably less than their methylated analogs or the ones that bear halides and hydrides [8,9]. This has implications in the limited application reactions of L_n_MCF_3_ (L = ligand) which are much more limited compared to the applications of the aforementioned analogs that include advances in C-H activation, C-C activation, small molecule activation, etc. [10,11]. An example of such a void in the literature of the much-studied dienyl metal complexes, is the fact that there are no cyclohexadienyl metal trifluoromethyl derivatives. This, while there is a plethora of report on methylated, hydride, halide analog complexes as well as interesting applications [10].

As a result, contributing to the synthetic chemistry of trifluoromethyl complexes, we investigated the synthesis of complexes with a cyclohexadienyl ligand. By using Morrison’s trifluoromethylating reagent, Cd(CF_3_)_2_(DME), [11] we prepared and fully characterized the (η^5^-C_6_H_7_)Fe(CO)_2_CF_3_ and we determined its structure by X-ray crystallography.

## 2. Experimental

All reactions and manipulations were performed in oven- or flame-dried glassware under Argon atmosphere either in a glovebox or by using standard Schlenk techniques. Column chromatography was carried out using silica gel 62 (60–200 mesh) supplied by Mallinckrodt SilicAR, which had been pre-dried at 250 °C under high vacuum. 1,2-dimethoxyethane (DME) and diethyl ether (Et_2_O) were distilled from sodium-benzophenone ketyl under nitrogen. Dichloromethane (CH_2_Cl_2_) was dried over calcium hydride (CaH_2_) and distilled under N_2_ prior to use. Copper bromide (CuBr) was purchased from Aldrich and dried by heating to 45 °C for 12 h under high vacuum. (C_6_H_7_)Fe(CO)_2_I, and Cd(CF_3_)_2_(DME) were prepared and purified by literature methods [12,13]. ^1^H NMR, [13] C NMR, and ^19^F NMR spectra were recorded on either a Bruker Avance 500, or a Bruker AM-400 spectrometer with Nalorac BB probes. All ^1^H chemical shifts (δ) are reported in ppm relative to trimethylsilane. ^19^F NMR chemical shifts are given in ppm downfield from CFCI_3_. Multiplicities are indicated by s (singlet), d (doublet). Coupling constants are reported in Hertz. Infrared spectra were recorded from KBr pellets on an ATI Mattson 106 Genesis II Series FT-IR spectrometer. Infrared bands are given in cm^−1^. Uncorrected melting points were determined with a Thomas Hoover capillary melting point apparatus in flame-sealed Pyrex capillaries. Low-resolution electron impact mass spectra (LR/MS-EI) and high-resolution electron impact mass spectra (HR/MS-EI) were obtained on a Finnigan LCQ spectrometer in the APCI mode. LRMS-EI were also obtained on a Hewlett Packard 5987A quadrupole instrument. Elemental combustion analyses were performed by Midwest Microlab, LLC of Indianapolis, IN. For the X-crystallographic analysis, a single crystal of (C_6_H_7_)Fe(CO)_2_CF_3_ was mounted on the goniometer of a Bruker three circle, single crystal diffractometer with APEX detector and SMART software, using an argon-filled capillary, and all data were collected at room temperature. All the experimental details of the crystallographic study are given in five tables in the Supplementary Material (Tables S1–S5).

**Synthesis of (C_6_H_7_)Fe(CO)_2_CF_3_ without CuBr.** C_6_H_7_Fe(CO)_2_l (50.0 mg, 0.157 mmol) and Cd(CF_3_)_2_(DME) (75 mg, 0.220 mmol) were placed into a 10 mL flask and the system was attached to a vacuum line. After evacuation, 3 mL of dry CH_2_Cl_2_ were distilled onto the reagents and the mixture was magnetically stirred at ambient temperature. After 5 h, the solution started to turn orange from brown. After 20 h, the color had changed to yellow. The solvent and all other volatile materials were removed under vacuum and a yellow solid was obtained. This solid was dissolved in 3 mL of Et_2_O and then chromatographed on a silica gel column under nitrogen, using Et_2_O as eluent. Upon removal of the solvent, a yellow solid later identified as (C_6_H_7_)Fe(CO)_2_CF_3_ (8.5 mg, 0.032 mmol) was obtained in 21% yield. The compound can also be purified by sublimation at ambient temperature under high vacuum (0.1 mm). It melts at 42–43 °C and it is air sensitive. ^1^H NMR (δ ppm in CDCl_3_): 2.11 (d, ^2^J_H-H_ = 14.1 Hz, 1H, C_6_**H**_7_), 2.79 (dt, ^2^J_H-H_ = 14.1 Hz, 1H, C_6_**H**_7_), 3.10 (t, ^3^J_H-H_ = 6.2 Hz, 2H, C_6_**H**_7_), 5.36 (t, ^3^J_H-H_ = 6.2 Hz, 2H, C_6_**H**_7_), 7.02 (t, ^3^J_H-H_ = 6.2 Hz, 1H, C_6_**H**_7_). ^13^C{^1^H} NMR (δ ppm in CDCl_3_): 23.7 (s, 2C, **C**_6_H_7_), 83.2 (s, 4C, **C**_6_H_7_). ^19^F NMR (δ ppm in CDCl_3_): −0.8 (s, 3F, C**F**_3_). FT-IR (cm^−1^, KBr pellet): 3090 (**C-H**), 2000 (**C-O**), 1996 (**C-O**), 1093 (**C-F**), 1044 (**C-F**), 991 (**C-F**). LRMS-EI (Ion, m/e, %): (C_6_H_7_)Fe(CO)_2_CF_3_ 260, 12; (C_6_H_7_)Fe(CO)CF_3_ 232, 2; (C_6_H_7_)Fe(CO)CF_2_ 213, 2; (C_6_H_7_)FeCF_2_ 185, 11; (C_6_H_6_)FeCO 162, 39; (C_6_H_7_)FeF 154, 36; (C_6_H_6_)Fe 134, 100; FeCF_3_ 125, 10. HRMS-EI m/z): found, 259.9738; Calcd. for C_9_H_7_F_3_FeO_2_, 259.9748 (Δ_m/m_ = 3.7 ppm). Elemental Analysis Found: C, 41.68; H, 2.88. Calcd. for C_9_H_7_F_3_FeO_2_: C, 41.58; H, 2.71.

**Synthesis of (C_6_H_7_)Fe(CO)_2_CF_3_ with CuBr.** C_6_H_7_Fe(CO)_2_l (150.0 mg, 0.472 mmol), Cd(CF_3_)_2_(DME) (200 mg, 0.588 mmol) and CuBr (200 mg, 1.394 mmol) were placed into a 25 mL flask and the system was attached to a vacuum line and cooled to −78 °C. After evacuation, 10 mL of dry dichloromethane were distilled onto the reagents and the mixture was warmed with stirring to room temperature. After 1 h, the solution started to turn orange from brown. Finally, after 3 h, the color had changed to yellow. The solvent and all other volatile materials were removed under vacuum and the yellow solid was obtained. This solid was dissolved in 5 mL of diethyl ether and then chromatographed on a silica gel column under nitrogen, using diethyl ether as eluent. Upon removal of the solvent, (C_6_H_7_)Fe(CO)_2_CF_3_ (87.1 mg, 0.335 mmol) was obtained in 71% yield. 

Supplementary data CCDC <2196136> contains the supplementary crystallographic data for the (C_6_H_7_)Fe(CO)_2_CF_3_ complex. These data can be obtained free of charge via http://www.ccdc.cam.ac.uk/conts/retrieving.html, accessed on 22 October 2022, or from the Cambridge Crystallographic Data Centre, 12 Union Road, Cambridge CB2 1EZ, UK; fax: (+44) 1223-336-033; or e-mail: deposit@ccdc.cam.ac.uk.

## 3. Results and Discussion

The main cause of the imbalance between the number of synthetic and application reports on trifluoromethylated metal complexes, L_n_M-CF_3_, and the reports on analogous complexes, L_n_M-CH_3_, L_n_M-H, and L_n_M-X (X = halide), is the lack of effective trifluoromethylating reagents as compared to the potency of analogous reagents for the other derivatives, which include, among others, alkyllithiums, Grignard in methylation reactions, sodium borohydride and lithium borohydride in hydride yielding reactions, and metal halides in preparing halide reactions [14]. This problem makes the introduction of the CF_3_ moiety to metal substrates complicated, especially when challenging substrates are subjected to trifluoromethylation [15]. An illustrative example is the organometallic chemistry of Fe, in which there is currently only one Fe-CF_3_ complex known in the literature in which the Fe center is connected to a organocyclic ligand. In contrast, there are hundreds of Fe-CH_3_, Fe-H and Fe-X counterparts reported. Moreover, X-ray analysis data on Fe-CF_3_ complexes is even more scarce in the literature with only a handful studied and published [16].

In our efforts to explore the synthetic chemistry; enrich the knowledge; and provide access to pathways that lead to trifluoromethyl compounds; this work has targeted the synthesis of the first trifluoromethylated cyclohexadienyl metal complex. Part of the interest in metal complexes bearing the cyclohexadienyl ligand is their potential in catalytic applications [17]. An iodo Fe precursor was selected as a substrate for nucleophilic trifluromethylation reactions. Upon surveying the literature of the limited number of previously synthesized Fe-CF_3_ containing complexes; we realized that apart of Morrison’s trifluoromethylating reagent; Cd(CF_3_)_2_(DME), which was utilized once in the past; only reactions with CF_3_I and decarboxylation reactions of a Fe-COCF_3_ bond; have been reported to result in formation of Fe-CF_3_ bonds [16,18]. A number of reactions were attempted utilizing milder nucleophilic trifluoromethylating reagents; but they did not result in Fe-CF_3_ bond formation at the various temperature and solvent conditions. In particular; as monitoring by ^19^F spectroscopy; the precursor (C_6_H_7_)Fe(CO)_2_I did not react for an iodo to trifluoromethyl exchange for all of the following previously utilized combinations at ambient and elevated temperatures: i. CF_3_I in CH_2_Cl_2_, [19] ii. AgCF_3_ prepared in situ from AgF and Ruppert’s reagent, [20] iii. Me_3_SiCF_3_ in THF, [21] and iv. CuCF_3_ prepared in situ from CF_3_CO_2_Na/CuI in NMP [22]. During these reactions; a variety of ^19^F NMR resonances; outside the M-CF_3_ region were observed, [23] most likely due to the nucleophilic attach of the CF_3_-moiety to the carbocylic ligand; consistent with reports of nucleophile; metal complex-assisted arene functionalization [24]. Attempts to isolate pure products on these reactions were unsuccessful.

**Figure 1:**
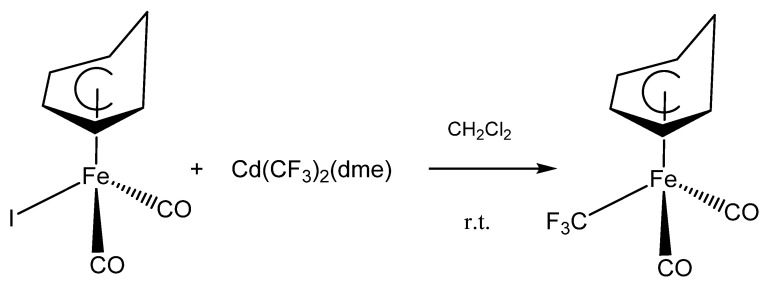
The reaction of (C_6_H_7_)Fe(CO)_2_CF_3_ with Morrison’s reagent, Cd(CF_3_)_2_(dme).

Upon treating (C_6_H_7_)Fe(CO)_2_I with Cd(CF_3_)_2_(DME) in anhydrous dichloromethane at ambient temperature; a slow reaction occurred that turned the brown solution yellow; converting the iodide to the new (C_6_H_7_)Fe(CO)_2_CF_3_ as shown in Figure 1. The progress of the reaction was monitored by ^19^F NMR spectroscopy which indicated the disappearance of the peak at −36.8 ppm (^2^J_F-Cd_ = 454 Hz); due to Cd(CF_3_)_2_(dme), [15] and the appearance of new signals at −0.8 and −0.2 ppm for (C_6_H_7_)Fe(CO)_2_CF_3_. These signals are consistent with the region previously reported for Fe-CF_3_ bonds [16,18]. As it has been observed for similar reactions; the yield of the yellow air-sensitive solids was enhanced by the addition of CuBr to the reaction mixture [15].

After purification the product were sublimed, to give highly pure (C_6_H_7_)Fe(CO)_2_CF_3_ as a yellow solid in 71% yield. The new complex is air sensitive decomposing in the air withing 48 h to a brown solid that has no ^19^F NMR signal. Moreover, the complex is moisture sensitive, decomposing upon exposure to wet solvent again to a solid that has no ^19^F NMR signal. The decomposition products were not investigated further. Finally, the new complex is not affected by light and it stays indefinitely stable in a flame sealed NMR tube.

The spectral properties of the new complex reveal five different protons in the ^1^H NMR with two distinct protons at 2.11 and 2.79 ppm for the perturbed saturated carbon, and three resonances for the remaining five protons of the five coplanar unsaturated carbons at 3.10, 5.36 and 7.02 ppm. The two saturated and unsaturated carbons also show two distinct resonances in the ^13^C NMR at 23.7 and 83.2 respectively. A singlet at −0.8 ppm is registered in the ^19^F NMR for the three equivalent fluorine atoms of the trifluoromethyl group, which in the same region as the −7.1 ppm of the Fe[(CF_3_)(CON^i^Pr_2_)(CO_2_)(PPh_3_)] and +10.2 ppm for the CpFe(CO)_2_CF_3_. Finally, the IR spectra of the new complex contained weak C-H stretches around 3100 cm^−1^ and two strong absorptions at 2000 and 1996 cm^−1^ from the symmetric and asymmetric C-O stretches of the carbonyl groups. The C-F stretches were observed as strong absorptions at 1093, 1044 and 991 cm^−1^. Comparison of the C-F stretching frequencies of the new complex with the ones at 1010 and 1055 cm^−1^ of the corresponding CpFe(CO)_2_CF_3_ may suggest that the cyclohexadienyl group is less electron donating than the cyclopentadienyl group, overall making the backbonding and the Fe-CF_3_ bond of the former weaker.

**Figure 2:**
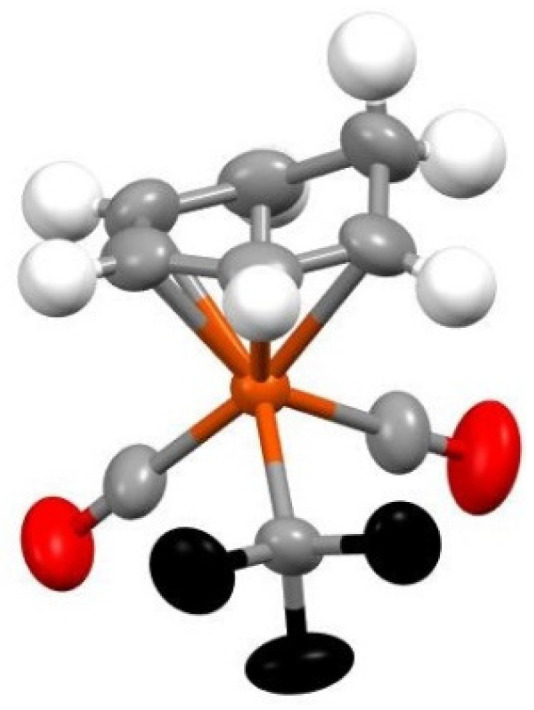
X-ray structure of (C_6_H_7_)Fe(CO)_2_CF_3_ at the 50% probability level.

Yellow crystals of the product were grown by layering anhydrous dichloromethane solution with anhydrous hexanes and keeping undisturbed for 14 days in a sealed NMR tube. This were analyzed by X-ray crystallography to give the structure of (C_6_H_7_)Fe(CO)_2_CF_3_ as shown in Figure 2.

In the analyzed structure and in line with what it has been reported before for structures of metal coordinated cyclohexadienyl organic rings [25], the six carbons are highly nonplanar, with one of them, the one and only saturated carbon being out of the plane that the remaining five carbon atoms form. Specifically, the methylene carbon lies ~0.63 Å above the unsaturated ring, and the Fe atom is 1.60 Å away from the plane of this ring, analogously to the corresponding values (~0.65 and 1.66 Å) reported by Shirin, et al. for a Ru-coordinated cyclohexadienyl ring [25]. The dihedral angle between the plane of the five carbons and the second plane of the three carbons that contains the saturated carbon is 45°, which is slightly larger but analogous to the 40° recorded for the (2-methoxycyclohexadienyl)Fe(CO)_3_^+^ species [26]. The Fe-CF_3_ bond of 1.968(3) Å, which very similar to the Fe-CF_3_ of one of the few iron trifluoromethyl structures Fe[(CF_3_)(CON^i^Pr_2_)(CO_2_)(PPh_3_)], which is 1.979(4) Å [16]. In general the trifluoromethyl group takes up almost identical positions with that of the Fe[(CF_3_)(CON^i^Pr_2_)(CO_2_)(PPh_3_)] complex, with the C-F bonds averaging 1.355(4) Å in the new complex vs. 1.353 Å in the old one and F-C-F angle 103.2° vs. 102.1°. Finally, the C-O bonds average is 1.225(4) Å, which is consistent with other Fe-CO bonds.

Hirshfeld surface analysis serves as a powerful tool for gaining additional insight into the intermolecular interaction of molecular crystals. The size and shape of Hirshfeld surface allows the qualitative and quantitative investigation and visualization of intermolecular close contacts in molecular crystals [27]. The Hirshfeld surface enclosing a molecule is defined by a set of points in 3D space where the contribution to the electron density from the molecule of interest is equal to the contribution from all other molecules. 

Molecular Hirshfeld surfaces are constructed based on electron distribution calculated as the sum of spherical atom electron densities [28]. Thus, an isosurface is obtained, and for each point of the isosurface two distances can be defined: *d*_e_, the distance from the point to the nearest atom outside to the surface, and *d*_i_, the distance to the nearest atom inside to the surface. Moreover, the identification of the regions of particular importance to intermolecular interactions is obtained by mapping normalized contact distance (*d*_norm_), expressed as:

*d*_norm_ = (*d*_i_ − *r*_i_^vdw^)/*r*_i_^vdw^ + (*d*_e_ − *r*_e_^vdw^)/*r*_e_^vdw^

where *r*_i_^vdw^ and *r*_e_^vdw^ are the van der Waals radii of the atoms [29]. The value of *d*_norm_ is negative or positive when intermolecular contacts are shorter or longer than *r*^vdw^, respectively. Graphical plots of the molecular Hirshfeld surfaces mapped with *d*_norm_ employ the red–white–blue color scheme where red color indicates the shorter intermolecular contacts, white color shows the contacts around the *r*^vdW^ separation, and blue color is used to indicate the longer contact distances. Because of the symmetry between *d*_e_ and *d*_i_ in the expression for *d*_norm_, where two Hirshfeld surfaces touch, both will display a red spot identical in color intensity as well as size and shape [30].

**Figure 3:**
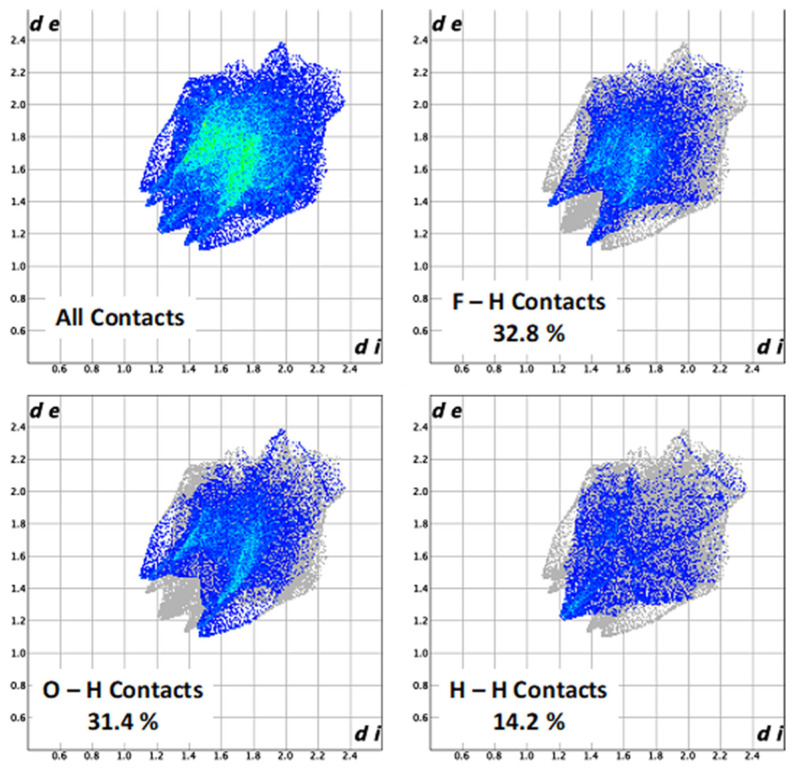
Fingerprint plots (*d*_e_ vs. *d*_i_) for the Hirshfeld surface of (C_6_H_7_)Fe(CO)_2_CF_3_. (**top left**) Full fingerprint plot; (**top right**) resolved fingerprint plot for the F–H contacts, accounting for 32.8% of the contacts; (**bottom left**) resolved fingerprint plot for the O–H contacts, accounting for 31.4% of the contacts; (**bottom right**) resolved fingerprint plot for the H–H contacts, accounting for 14.2% of the contacts.

The combination of *d*_e_ and *d*_i_ in the form of a 2D fingerprint plot provides summary of intermolecular contacts in the crystal and are in complement to the Hirshfeld surfaces [31]. Such plots provide information about the intermolecular interactions in the immediate environment of each molecule in the asymmetric unit. Moreover, the close contacts between particular atom types can be highlighted in so-called resolved fingerprint plots, [29] which allows the facile assignment of an intermolecular contact to a certain type of interaction and quantitatively summarize the nature and type of intermolecular contacts. The Hirshfeld surfaces are mapped with *d*_norm_, *d*_e_, and 2D fingerprint plots presented were generated using Crystal-Explorer 17.5 [32,33,34,35,36]. As can be seen from the results of the contact analysis, presented in Figure 3 and summarized in Table 1, H-X interactions account for 84.8% of all contacts, signifying a highly compact solid-state structure. 

## 4. Conclusions

Filling a void in the chemistry literature, and facilitating access to novel trifluoromethyl metal complexes, the synthesis and full characterization, including X-ray diffraction of the first cyclohexadienyl metal complexes (C_6_H_7_)Fe(CO)_2_CF_3_ in high yields is reported by the reaction of the iron precursor with Morrison’s trifluoromethylating reagent, Cd(CF_3_)_2_(DME) at room temperature. The new complex is an air- and moisture-sensitive complex species that was prepared in 71% yield. X-ray crystallography of the new complex revealed that the six carbons are highly nonplanar, with one of them, the one and only saturated carbon being out of the plane that the remaining five carbon atoms form, while the Fe-CF_3_ bond is very similar to Fe-CF_3_ bonds reported in the past. Finally, Hirshfeld surface analysis was applied to gain additional understanding into the intermolecular interaction of iron crystal. According to the results of the analysis H-X interactions account for 84.8% of all contacts, indicating a highly compact solid-state structure.

## Figures and Tables

**Table 1:** Hirshfeld surface analysis results for complex (C_6_H_7_)Fe(CO)_2_CF_3_.

Contact	Percent of Total Contacts	Contact	Percent of Total Contacts
F–H	32.8%	F–C	3.9%
O–H	31.4%	O–O	1.7%
H–H	14.2%	O–C	1.3%
F–O	8.2%	C–C	0%
C–H	6.4%		

## Data Availability

This is not applicable.

## Supplementary Materials

The following supporting information can be downloaded at: https://www.mdpi.com/article/10.3390/molecules27217595/s1. Figure S1: ^19^F NMR of (C_6_H_7_)Fe(CO)_2_CF_3_; Table S1: Crystal data and structure refinement; Table S2: Bond Lengths [Å]; Table S3: Bond Angles (°); Table S4: Anisotropic displacement parameters (Å^2^ × 10^3^) The anisotropic displacement factor exponent takes the form: -2p^2^[h^2^ a*^2^U^11^ + … + 2 h k a* b* U^12^]; Table S5: Atomic coordinates (×10^4^) and equivalent isotropic displacement parameters (Å^2^ × 10^3^) for cdwjm81m. U(eq) is defined as one third of the trace of the orthogonalized U^ij^ tensor.
