# Supplementary material for: Synthesis, Characterization, and X-ray Crystallography, of the First Cyclohexadienyl Trifluoromethyl Metal Complex (η5-C6H7)Fe(CO)2CF3
Source: Molecules. 2022 Nov 5;27(21):7595. doi: 10.3390/molecules27217595 (PMC9654653; doi:10.3390/molecules27217595)

Synthesis, Characterization, and X-ray Crystallography, of the first Cyclohexadienyl Trifluoromethyl Metal Complex (C6H7)Fe(CO)2CF3

Chris Douvris [1*], David Matatov [1], Derek Bussan [2], Christos Lampropoulos [3,4,5] and Donald J. Wink [5]

[1] Department of Biological and Chemical Sciences, New York Institute of Technology, Old Westbury, NY 11564, USA
[2] Department of Chemistry, Eastern Kentucky University, 521 Lancaster Ave, Richmond, KY 40475, USA
[3] Florida State College at Jacksonville, Jacksonville, FL, 32202, USA
[4] James Weldon Johnson MS, Duval County Public Schools, Jacksonville, FL 32207, USA
[5] Department of Chemistry, University of Illinois, Chicago, 845 W. Taylor St., Chicago, IL 60607, USA

Experimental Details for Crystallographic Study of $(C_6H_7)Fe(CO)_2CF_3$.

Table S1. Crystal data and structure refinement.

Empirical formula	C9 H7 F3 Fe O2	
Formula weight	260.00	
Temperature	571(2) K	
Wavelength	0.71073 Å	
Crystal system	ORTHORHOMBIC P-lattice	
Space group	P2(1)2(1)2(1)	
Unit cell dimensions	a = 7.6435(11) Å	a= 90°.
	b = 7.8718(11) Å	b= 90°.
	c = 16.042(2) Å	g = 90°.
Volume	965.217(17) $Å^3$	
Z	4	
Density (calculated)	1.786 Mg/m^3	
Absorption coefficient	1.579 mm^{-1}	
F(000)	520	
Theta range for data collection	2.54 to 28.21°.	
Index ranges	-9<=h<=10, -10<=k<=10, -21<=l<=21	
Reflections collected	8996	
Independent reflections	2293 [R(int) = 0.0785]	
Completeness to theta = 28.21°	98.2 %	
Refinement method	Full-matrix least-squares on F^2	
Data / restraints / parameters	2293 / 0 / 136	
Goodness-of-fit on F^2	0.999	
Final R indices [I>2sigma(I)]	R1 = 0.0445, wR2 = 0.1037	
R indices (all data)	R1 = 0.0549, wR2 = 0.1082	
Absolute structure parameter	0.36(3)	
Largest diff. peak and hole	0.510 and -0.386 e.$Å^{-3}$	

Table S2. Bond Lengths [Å]

Number	Atom1	Atom2	Type	Polymeric	Cyclicity	Length	SybylType
1	Fe1	C1	Unknown	no	cyclic	2.084(4)	1
1	C1	Fe1	Unknown	no	cyclic	2.084(4)	1
2	Fe1	C2	Unknown	no	cyclic	2.095(4)	1
2	C2	Fe1	Unknown	no	cyclic	2.095(4)	1
3	Fe1	C3	Unknown	no	cyclic	2.155(3)	1
3	C3	Fe1	Unknown	no	cyclic	2.155(3)	1
4	Fe1	C5	Unknown	no	cyclic	2.211(4)	1
4	C5	Fe1	Unknown	no	cyclic	2.211(4)	1
5	Fe1	C6	Unknown	no	cyclic	2.130(3)	1
5	C6	Fe1	Unknown	no	cyclic	2.130(3)	1
6	Fe1	C7	Unknown	no	acyclic	1.968(3)	1
6	C7	Fe1	Unknown	no	acyclic	1.968(3)	1
7	Fe1	C8	Unknown	no	acyclic	1.784(3)	un
7	C8	Fe1	Unknown	no	acyclic	1.784(3)	un
8	Fe1	C9	Unknown	no	acyclic	1.781(3)	un
8	C9	Fe1	Unknown	no	acyclic	1.781(3)	un
9	F7A	C7	Unknown	no	acyclic	1.357(4)	1
9	C7	F7A	Unknown	no	acyclic	1.357(4)	1
10	F7B	C7	Unknown	no	acyclic	1.356(5)	1
10	C7	F7B	Unknown	no	acyclic	1.356(5)	1
11	F7C	C7	Unknown	no	acyclic	1.352(4)	1
11	C7	F7C	Unknown	no	acyclic	1.352(4)	1
12	O8	C8	Unknown	no	acyclic	1.117(4)	un
12	C8	O8	Unknown	no	acyclic	1.117(4)	un
13	O9	C9	Unknown	no	acyclic	1.128(4)	un
13	C9	O9	Unknown	no	acyclic	1.128(4)	un
14	C1	H7	Unknown	no	acyclic	0.931	1
14	H7	C1	Unknown	no	acyclic	0.931	1
15	C1	C2	Unknown	no	cyclic	1.406(5)	1
15	C2	C1	Unknown	no	cyclic	1.406(5)	1
16	C1	C6	Unknown	no	cyclic	1.404(6)	1
16	C6	C1	Unknown	no	cyclic	1.404(6)	1
17	C2	H6	Unknown	no	acyclic	0.930	1
17	H6	C2	Unknown	no	acyclic	0.930	1
18	C2	C3	Unknown	no	cyclic	1.377(6)	1
18	C3	C2	Unknown	no	cyclic	1.377(6)	1
19	C3	H1	Unknown	no	acyclic	0.930	1
19	H1	C3	Unknown	no	acyclic	0.930	1
20	C3	C4	Unknown	no	cyclic	1.481(6)	1
20	C4	C3	Unknown	no	cyclic	1.481(6)	1
21	C4	H4	Unknown	no	acyclic	0.970	1
21	H4	C4	Unknown	no	acyclic	0.970	1
22	C4	H5	Unknown	no	acyclic	0.970	1
22	H5	C4	Unknown	no	acyclic	0.970	1
23	C4	C5	Unknown	no	cyclic	1.507(7)	1

23	C5	C4	Unknown	no	cyclic	1.507(7)	1
24	C5	H3	Unknown	no	acyclic	0.929	1
24	H3	C5	Unknown	no	acyclic	0.929	1
25	C5	C6	Unknown	no	cyclic	1.361(7)	1
25	C6	C5	Unknown	no	cyclic	1.361(7)	1
26	C6	H2	Unknown	no	acyclic	0.930	1
26	H2	C6	Unknown	no	acyclic	0.930	1

Table S3. Bond Angles (°)

Number	Atom1	Atom2	Atom3	Angle
1	C1	Fe1	C2	39.3(1)
2	C1	Fe1	C3	69.0(1)
3	C1	Fe1	C5	67.9(2)
4	C1	Fe1	C6	38.9(2)
5	C1	Fe1	C7	107.0(1)
6	C1	Fe1	C8	155.7(2)
7	C1	Fe1	C9	99.2(2)
8	C2	Fe1	C3	37.8(1)
9	C2	Fe1	C5	79.4(1)
10	C2	Fe1	C6	69.4(1)
11	C2	Fe1	C7	87.7(1)
12	C2	Fe1	C8	128.6(1)
13	C2	Fe1	C9	133.2(2)
14	C3	Fe1	C5	65.1(2)
15	C3	Fe1	C6	79.3(2)
16	C3	Fe1	C7	99.4(1)
17	C3	Fe1	C8	92.4(2)
18	C3	Fe1	C9	167.7(2)
19	C5	Fe1	C6	36.5(2)
20	C5	Fe1	C7	164.5(2)
21	C5	Fe1	C8	90.4(2)
22	C5	Fe1	C9	107.9(2)
23	C6	Fe1	C7	144.4(2)
24	C6	Fe1	C8	124.7(2)
25	C6	Fe1	C9	89.3(2)
26	C7	Fe1	C8	90.9(1)
27	C7	Fe1	C9	87.2(2)
28	C8	Fe1	C9	98.0(2)
29	Fe1	C1	H7	127.7
30	Fe1	C1	C2	70.7(2)
31	Fe1	C1	C6	72.3(2)
32	H7	C1	C2	121.2
33	H7	C1	C6	121.1
34	C2	C1	C6	117.7(3)
35	Fe1	C2	C1	69.9(2)
36	Fe1	C2	H6	128.4
37	Fe1	C2	C3	73.5(2)

38	C1	C2	H6	120.3
39	C1	C2	C3	119.4(3)
40	H6	C2	C3	120.3
41	Fe1	C3	C2	68.7(2)
42	Fe1	C3	H1	107.2
43	Fe1	C3	C4	94.0(3)
44	C2	C3	H1	119.7
45	C2	C3	C4	120.7(4)
46	H1	C3	C4	119.6
47	C3	C4	H4	111.0
48	C3	C4	H5	111.0
49	C3	C4	C5	103.8(4)
50	H4	C4	H5	109.0
51	H4	C4	C5	111.0
52	H5	C4	C5	111.0
53	Fe1	C5	C4	91.0(3)
54	Fe1	C5	H3	110.4
55	Fe1	C5	C6	68.5(3)
56	C4	C5	H3	120.0
57	C4	C5	C6	120.0(4)
58	H3	C5	C6	120.0
59	Fe1	C6	C1	68.8(2)
60	Fe1	C6	C5	75.0(3)
61	Fe1	C6	H2	128.8
62	C1	C6	C5	120.6(4)
63	C1	C6	H2	119.7
64	C5	C6	H2	119.7
65	Fe1	C7	F7A	115.1(2)
66	Fe1	C7	F7B	115.1(2)
67	Fe1	C7	F7C	115.5(2)
68	F7A	C7	F7B	103.2(3)
69	F7A	C7	F7C	103.4(3)
70	F7B	C7	F7C	102.9(3)
71	Fe1	C8	O8	178.3(3)
72	Fe1	C9	O9	178.3(4)

Table S4. Anisotropic displacement parameters (Å^{2}x 10^3) The anisotropic displacement factor exponent takes the form: -2p^2[h^2 a*^{2}U^{11} + ... + 2 h k a* b* U^{12}]

	U^{11}	U^{22}	U^{33}	U^{23}	U^{13}	U^{12}
Fe1	39(1)	36(1)	38(1)	-1(1)	4(1)	2(1)
C5	71(3)	38(2)	82(3)	9(2)	10(3)	10(2)
F7A	130(3)	47(1)	98(2)	13(2)	31(2)	-6(2)
F7C	117(3)	49(1)	71(2)	-17(1)	-20(2)	12(2)

C9	51(2)	65(3)	45(2)	-2(2)	2(2)	-13(2)
F7B	76(2)	67(2)	116(3)	-5(2)	-28(2)	32(2)
C3	76(4)	44(2)	67(3)	4(2)	1(3)	-6(2)
C1	44(2)	49(2)	70(3)	10(2)	6(2)	-7(2)
C8	51(3)	73(3)	52(2)	-7(2)	1(2)	10(2)
C4	82(4)	55(2)	44(2)	12(2)	8(2)	4(2)
O9	98(3)	121(3)	43(2)	-1(2)	0(2)	-21(3)
C2	63(3)	55(3)	51(2)	8(2)	24(2)	1(2)
C4	81(4)	63(3)	84(4)	6(3)	-19(3)	18(3)
O8	46(2)	119(3)	105(3)	-24(2)	-6(2)	-5(2)
C7	62(3)	39(2)	53(2)	7(2)	-3(2)	-1(2)

Table S5. Atomic coordinates (x 10^4) and equivalent isotropic displacement parameters (Å^{2}x 10^3) for cdwjm81m. U(eq) is defined as one third of the trace of the orthogonalized U^{ij} tensor.

	x	y	z	U(eq)
Fe1	7836(1)	5789(1)	3513(1)	38(1)
C5	8395(7)	3107(6)	3812(3)	63(1)
F7B	8130(5)	9231(4)	3028(2)	92(1)
F7C	7504(4)	8962(3)	4307(2)	79(1)
C8	7672(6)	5970(6)	2415(2)	54(1)
F7A	5552(4)	8597(4)	3385(2)	86(1)
C6	6778(7)	3292(6)	3428(3)	62(1)
C1	5522(6)	4459(5)	3748(3)	54(1)
C9	10095(6)	6253(6)	3611(3)	59(1)
C3	7654(7)	5188(6)	4819(3)	60(1)
O8	7597(5)	6102(6)	1707(2)	88(1)
C2	5977(6)	5398(6)	4454(3)	57(1)
C4	8563(8)	3541(7)	4736(3)	76(2)
O9	11558(5)	6525(6)	3676(3)	90(1)
C7	7239(6)	8215(5)	3561(3)	51(1)

Figure S1. ^{19}F NMR of $(C_6H_7)Fe(CO)_2CF_3$.

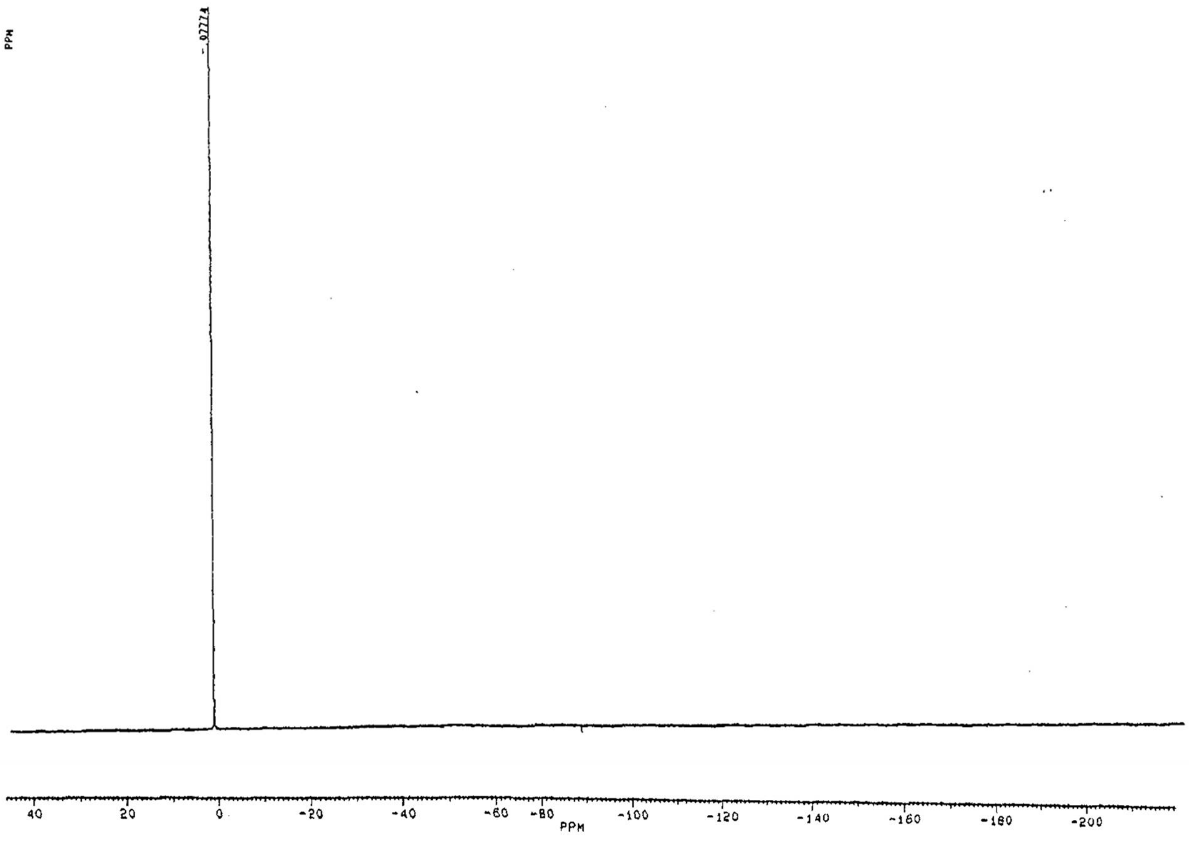

Supplement: Supplementary file 1 [file molecules-27-07595-s001.zip › molecules-1889152-supplementary.pdf]
